# Estimation of Brachial-Ankle Pulse Wave Velocity With Hierarchical Regression Model From Wrist Photoplethysmography and Electrocardiographic Signals: Method Design

**DOI:** 10.2196/58756

**Published:** 2025-08-26

**Authors:** Chih-I Ho, Chia-Hsiang Yen, Yu-Chuan Li, Chiu-Hua Huang, Jia-Wei Guo, Pei-Yun Tsai, Hung-Ju Lin, Tzung-Dau Wang

**Affiliations:** 1 Department of Electrical Engineering, National Central University, Taoyuan, Taiwan; 2 Graduate School of Advanced Technology, National Taiwan University, Taipei, Taiwan; 3 Cardiovascular Center and Divisions of Cardiology and Hospital Medicine, Department of Internal Medicine, National Taiwan University Hospital, No.7, Chung Shan S Rd, Taipei, 100225, Taiwan, 886 2-2312-3456

**Keywords:** photoplethysmography, PPG, pulse wave velocity, brachial-ankle pulse wave velocity, XGBoost, electrocardiography, signal processing, random forest

## Abstract

**Background:**

Photoplethysmography (PPG) signals captured by wearable devices can provide vascular age information and support pervasive and long-term monitoring of personal health condition.

**Objective:**

In this study, we aimed to estimate brachial-ankle pulse wave velocity (baPWV) from wrist PPG and electrocardiography (ECG) from smartwatch.

**Methods:**

A total of 914 wrist PPG and ECG sequences and 278 baPWV measurements were collected via the smartwatch from 80 men and 82 women with average age of 63.4 (SD 13.4) and 64.3 (SD 11.6) years. Feature extraction and weighted pulse decomposition were applied to identify morphological characteristics regarding blood volume change and component waves in preprocessed PPG and ECG signals. A systematic strategy of feature combination was performed. The hierarchical regression method based on the random forest for classification and extreme gradient boosting (XGBoost) algorithms for regression was used, which first classified the data into subdivisions. The respective regression model for the subdivision was constructed with an overlapping zone.

**Results:**

By using 914 sets of wrist PPG and ECG signals for baPWV estimation, the hierarchical regression model with 2 subdivisions and an overlapping zone of 400 cm per second achieved root-mean-square error of 145.0 cm per second and 141.4 cm per second for 24 men and 26 women, respectively, which is better than the general XGBoost regression model and the multivariable regression model (all *P*<.001).

**Conclusions:**

We for the first time demonstrated that baPWV could be reliably estimated by the wrist PPG and ECG signals measured by the wearable device. Whether our algorithm could be applied clinically needs further verification.

## Introduction

Cardiovascular disease (CVD) is a major cause of death and disability globally. Hemodynamic parameters are essential to the assessment of CVD risks. Arterial compliance is defined as the change of arterial blood volume for a given change in pressure and reflects the extent of arterial stiffness. Pulse wave velocity (PWV) describes the propagation of pulsatile activity due to ventricular ejection of blood and its interaction with arterial compliance [1]. Carotid-femoral PWV (cfPWV) and brachial-ankle PWV (baPWV) are associated with future CVD risk and commonly measured for clinic use. Compared with cfPWV, baPWV can be easily obtained by the oscillometric method with cuffs on the 4 limbs and is more widely used [2].

Owing to the advance of technology, wearable devices with automatic or self-assisted monitoring have been recognized as a promising tool to facilitate the assessment and management of CVD risks. Photoplethysmography (PPG) [3,4], ballistocardiography [5,6], electrical bioimpedance [7], or tonometry [8] has been widely studied for these purposes. Due to the ease of implementation, the optical PPG module is more often integrated into the wearable devices. The potential of estimation of BP [9,10] and PWV [11-13] from PPG signals attracts much attention.

Various approaches have been investigated to estimate PWV from PPG signals of different measurement sites [14]. The contour of PPG and its associated time interval features have been used to estimate either baPWV or cfPWV by approaches including multiple regression, artificial neural network, and support vector machine [15,16]. Most of the prior works used finger PPG signals for PWV estimation because of its clear contour and ease of feature extraction, compared with wrist PPG [17,18]. However, with the growing popularity of smartwatches as wearable health care devices, the use of wrist-based PPG in biomedical applications has attracted considerable attention. In this study, we aimed to estimate baPWV from wrist PPG and electrocardiography (ECG).

## Methods

Methods and statistical analysis are briefly summarized in this section. Further details are provided in the Supplementary Section.

### Data Collection

**Figure 1.**
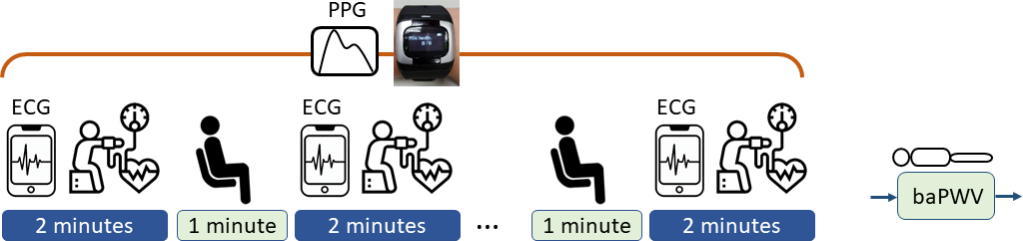
Measurement flow. baPWV: brachial-ankle pulse wave velocity; ECG: electrocardiography; PPG: photoplethysmography.

Figure 1 shows the measurement flow. Each volunteer wore a SENSIO smartwatch recording wrist PPG and ECG during the experimental period. For volunteers in the health management center, 3 rounds of measurements were conducted. For volunteers in the outpatient clinic, 5 rounds of measurements were made. In each round, the participants maintained the sitting position, and ECG was measured in the first minute. Blood pressures were then measured by the sphygmomanometer on the other arm (not wearing the smartwatch) with the cuff aligned at the heart level. A one-minute rest was reserved between 2 adjacent rounds. The wrist PPG signals were continuously recorded throughout the course. In the end, baPWV was measured by the OMRON noninvasive vascular screening device, with the cuffs on 4 limbs in the supine position.

### Ethical Considerations

The experiment was approved by the research ethics committee of National Taiwan University Hospital (number 201902087RIPA). All data were collected in accordance with the approved protocol. Importantly, the dataset used in this study did not contain any personally identifiable information, and all records were fully anonymized prior to analysis. Informed consent was obtained from all participants, and the study was conducted in compliance with the ethical standards set forth in the Declaration of Helsinki and relevant national regulations.

### Processing Flow

**Figure 2.**
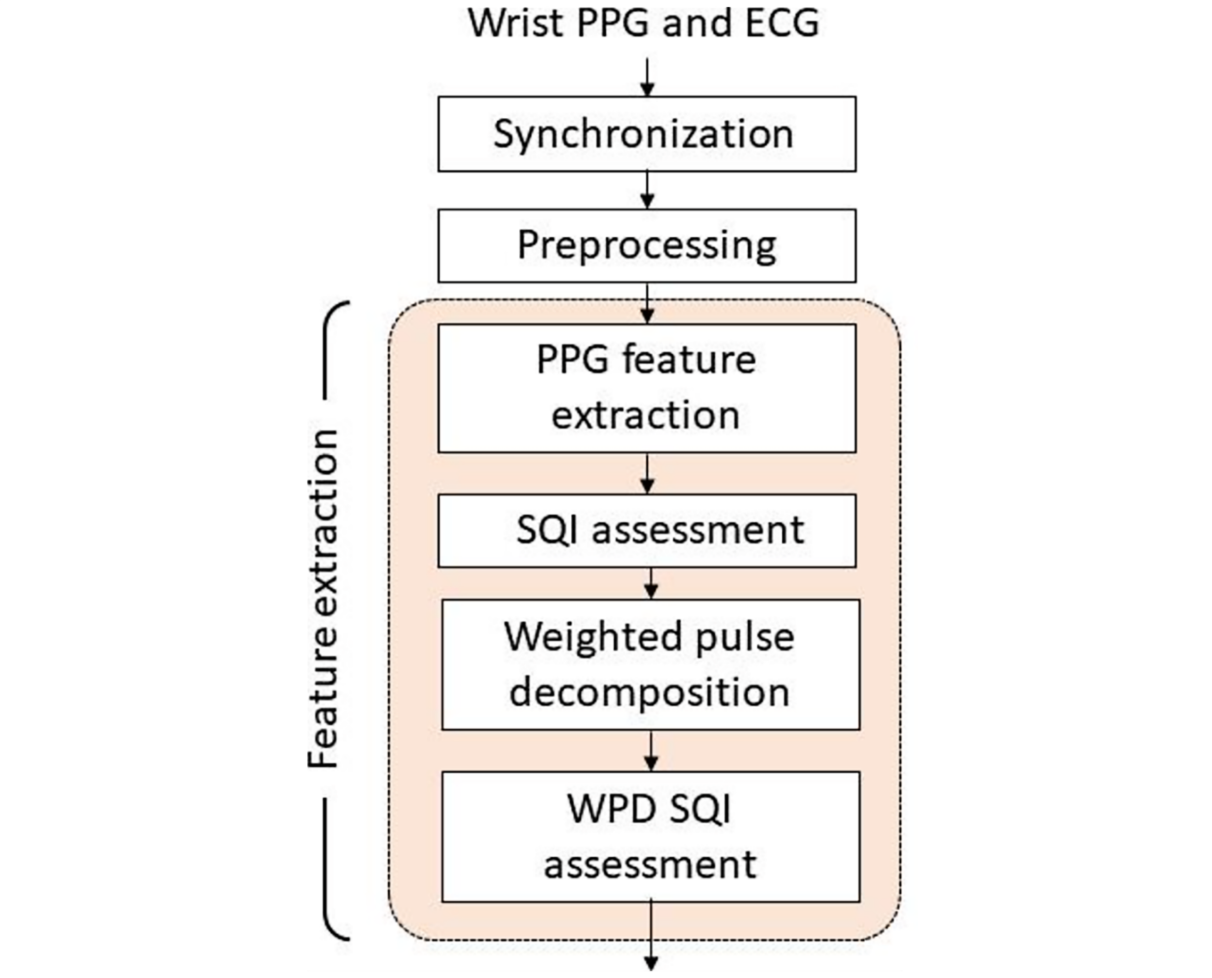
Signal-processing flow. ECG: electrocardiography; PPG: photoplethysmography; SQI: signal quality index; WPD: weighted pulse decomposition.

**Figure 3.**
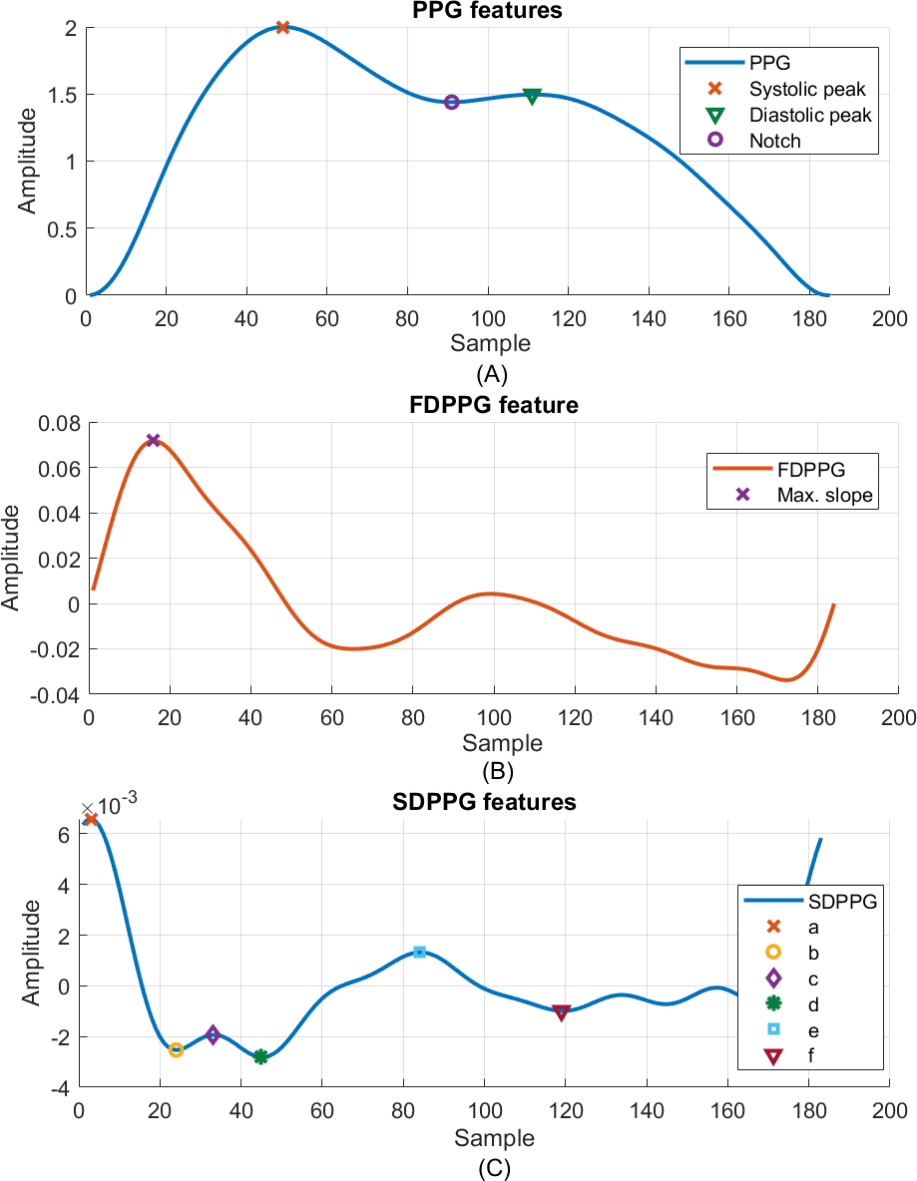
Photoplethysmography, first-order derivative photoplethysmography, and second-order derivative photoplethysmography waveforms and features in 1 cardiac cycle (from A to C). FDPPG: first-order derivative photoplethysmography; PPG: photoplethysmography; SDPPG: second-order derivative photoplethysmography.

The signal-processing flow is indicated in Figure 2. The PPG and ECG, sampled at 256 Hz, were extracted from the first minute of each round in the synchronization phase (Figures S1 A and S1 B in Multimedia Appendix 1). In the preprocessing phase, baseline wandering of signals was corrected by the discrete wavelet transform, and the 60-Hz power interference was suppressed by the notch filter. The amplitude of the whole signal segment was then normalized to [−1, +1]. The R peak of ECG and the valley of PPG signals were detected to calculate cycle length (Figures S1 C and S1 D in Multimedia Appendix 1). The skewness and variation of ECG and PPG cycle lengths were adopted to establish the signal quality index to exclude suboptimal ECG or PPG cycles for feature extraction. The first-order derivative PPG (FDPPG) and the second-order derivative PPG (SDPPG) signals were calculated. The systolic peak, notch, and diastolic peak were marked by the algorithm [19] for each PPG cycle (Figure 3A). The maximal slope (max slope) of the ascending systolic pulse, corresponding to the maximal rate of blood volume change, was identified by the first local maximum in FDPPG (Figure 3B) [20]. The local extrema of the SDPPG in systole are defined as a, b, c, and d points, where points a and c are local maxima and points b and d are local minima (Figure 3C) [21]. Point e is the local maximum around the boundary of systole and diastole in SDPPG. Point f is the first local minimum after point e.

The PPG pulse is regarded as a summation of several component waves, including the forward waves by left ventricular contraction and the distally reflected waves due to aortic elasticity and reservoir property [22]. The pulse decomposition analysis helps segregate the component waves [23]. With proper weighting, the variation of component waves can be reduced [24]. Five Gaussian waves are used for synthesizing the PPG pulse. Given $\theta_{i}=\left\{\alpha_{i},\beta_{i},\gamma_{i}\right\}$ corresponding to pulse amplitude, pulse position, and pulse width of the component wave i, and $\Theta =\left\{\theta_{1},\;\theta_{2},\;\ldots ,\theta_{5}\right\}$, the summation of the Gaussian waves takes the form of


(1) $$G(t|\theta )=\sum_{i=1}^{5}g(t|\theta_{i})$$

with


(2) $$g\left(t|\theta_{i}\right)=\alpha_{i}e^{\frac{{\left(t-\beta_{i}T_{s}\right)}^{2}}{2{\left(\gamma_{i}T_{s}\right)}^{2}}}$$

Denote *G_i_* as the component wave described by $g\left(t|\theta_{i}\right)$. Given the boundary constraints, $L_{\alpha_{i}}\leq \alpha_{i}\leq U_{\alpha_{i}}$, $L_{\beta_{i}}\leq \beta_{i}\leq U_{\beta_{i}}$, and $L_{\gamma_{i}}\leq \gamma_{i}\leq U_{\gamma_{i}}$ [24], the interior-point method is used to solve the following optimization problem,


(3) $$\hat{\Theta }=\arg\min_{\Theta }\frac{1}{M}\sum_{n=1}^{M}w\left(n\right)\left[s\left(n\right)-G\left(nT_{s}|\Theta \right)\right],$$

where w(n) is the weight to emphasize the informative portion of the PPG pulse $s\left(n\right)$ with length M and is given by


(4) $$w\left(n\right)=\left\{ \begin{array}{lc} \omega & n_{a}\leq n\leq n_{f}\\ 1 & else \end{array}\right.$$

Variables $n_{a}$ and $n_{f}$ refer to the position of points a and f. The weight ω is set to 80 for stabilizing the variation of component waves in the sequence with acceptable mean square error between the synthesized waveform and original waveform.

**Figure 4.**
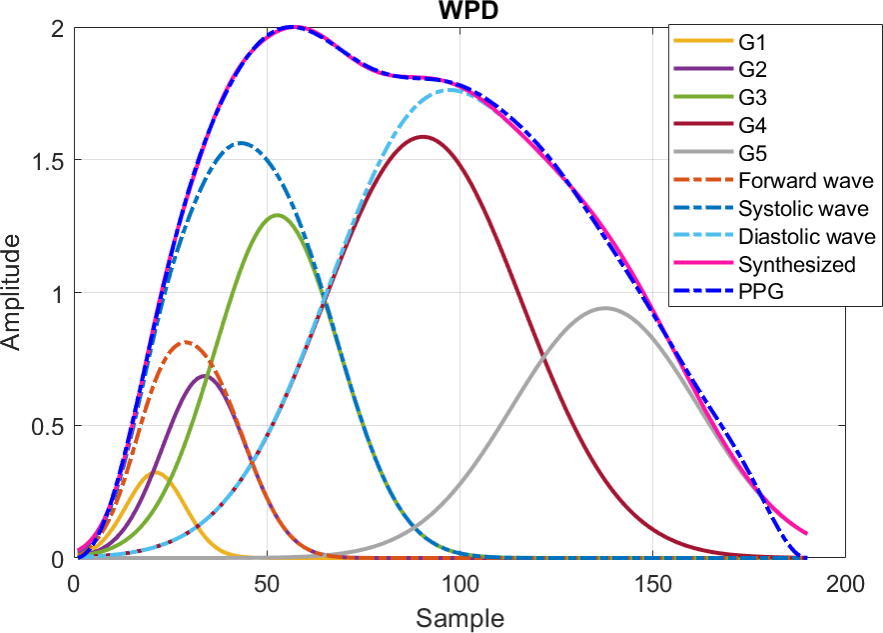
Component waves after weighted pulse decomposition. G1: Gaussian component wave 1; G2: Gaussian component wave 2; G3: Gaussian component wave 3; G4: Gaussian component wave 4; G5: Gaussian component wave 5; WPD: weighted pulse decomposition.

Once the component waves are acquired, the forward wave is generated by combining *G_1_* and *G_2_*. The systolic wave and diastolic wave are derived by combining *G_1_* to *G_3_* and *G_4_* to *G_5_*, respectively. The respective peaks of the synthesized forward wave, systolic wave, and diastolic wave are named as $p_{f}$, $p_{s}$, and $p_{d}$. In the following, the amplitude and position of feature x in the PPG pulse are indicated by $A_{x}$ and $n_{x}$, respectively. The amplitude of feature x in the ith-order derivative PPG is represented by$A_{x}^{\left(i\right)}$. The result of decomposed component waves by weighted pulse decomposition (WPD) is shown in Figure 4.

To assess the quality of WPD, WPD signal quality index, which was defined as mean square error between the PPG pulse, s(n), and the synthesized pulse, $G\left(nT_{s}|\Theta \right)$, of >$2\times 10^{-3}$, was implemented to remove disqualified pulses.

A total of 22 features were derived from the PPG pulse, FDPPG, and SDPPG (Table S1 A in Multimedia Appendix 2). The age index, which has been shown to be correlated with the augmentation index of aortic pressure [21,25],


(5) $$\frac{A_{b}^{\left(2\right)}-A_{c}^{\left(2\right)}-A_{d}^{\left(2\right)}-A_{e}^{\left(2\right)}}{A_{a}^{\left(2\right)}}$$

and its related variant combining only highly correlated components,


(6) $$\frac{A_{b}^{\left(2\right)}-A_{c}^{\left(2\right)}-A_{d}^{\left(2\right)}}{A_{a}^{\left(2\right)}}$$

were also used. There were 27 features derived from WPD (Table S1 B in Multimedia Appendix 2). The stiffness index (SI) is defined as the time interval between the peaks of systolic and diastolic waves [23] and is denoted by $n_{p_{d}}-n_{p_{s}}$. The time intervals of the third or fourth component wave to the forward wave were also calculated. Note that $n_{p_{s}}$ and $n_{p_{d}}$ were obtained from synthesized systolic wave peak $p_{s}$ and diastolic wave peak $p_{d}$ of WPD as shown in Figure 4 while $n_{sys}$ and $n_{dia}$ were marked as the positions of systolic peak and diastolic peak in PPG as shown in Figure 3.

The ECG-related features were also adopted (Table S1 C in Multimedia Appendix 2). The R peak and T peak of the ECG waveform were identified and marked as $n_{R}$ and $n_{T}$. Since the R peak occurs earlier than the PPG valley of the same heartbeat, $n_{R}$ is negative in number. The pulse arrival time (PAT) measures the time span between R peak and PPG valley, denoted by $-n_{R}$. PAT^2^ and Height^2^/PAT^2^ were included since either linear or nonlinear relationship between BP and pulse transit time has been shown [26]. The time span from R peak to maximum slope, peak of systolic wave, or component wave 2 was also considered.

Basic information (Table S1 D in Multimedia Appendix 2) contains age, height (H), weight, BMI, and lengths from arm to wrist $\left(L_{aw}\right)$ and finger $\left(L_{af}\right)$. The lengths from heart to brachium $\left(L_{b}\right)$ and from heart to ankle $\left(L_{a}\right)$ can be approximated by [27]


(7) $$L_{a}=0.8219H+12.328$$

(8) $$L_{b}=0.2195H-2.073.$$

The length difference between ankle and brachium could be expressed by $L_{a}-L_{b}$.

Feature normalization is often adopted since the relative change of 2 features could provide additional information than each feature alone. To systematically derive the normalization results, we generate combined features by dividing the value of feature u by value of feature v. The combined features contain not only magnitude-normalized or time-normalized features but also basic information features.

### Estimation Approach

#### Multivariable Regression

Linear regression and multivariable regression had been applied for baPWV estimation [12,28]. The time difference between the systolic peak to diastolic peak has been used and normalized by the Fridericia formula [28] while the systolic peak to the next onset (P2O), $M-n_{sys}$ (feature 1 in Table S1 A in Multimedia Appendix 2), of the PPG signal normalized by the PPG pulse length was also examined for PWV estimation [12]. These 2 variables were selected from the finger PPG features by the authors due to their high correlation to baPWV reported in the literature. The wrist PPG was used in this study for baPWV estimation. Because diastolic peak often vanished in wrist PPG pulses, we used SI (feature 51 in Table S1 B in Multimedia Appendix 2), which denotes the time span between peaks of decomposed systolic wave and diastolic wave according to WPD, and its normalized form with the Friderician formula is given by $SI/M^{1/3}$. The multivariable linear equations are described by [12,28]


(9) $$PWV=C_{1}Age+C_{2}\frac{SI}{M^{1/3}}+C_{3}$$

and


(10) $$PWV=C_{1}Age+C_{2}\frac{P2O}{M}+C_{3}.$$

#### Hierarchical Regression

The linear estimation regarding the correlations between PPG features and PWV, as used in multivariable regression analysis, may oversimplify the vascular hemodynamic state. The machine learning algorithms have been prosperously developed and used for biomedical applications, such as neural network and decision tree regression for estimation of vascular age [29] and gradient boosting decision tree regression for estimation of blood pressure [30]. We herein developed the hierarchical regression model based on the random forest and extreme gradient boosting (XGBoost) algorithms. A general regression model by XGBoost was also implemented for comparison.

The random forest and XGBoost algorithms of high scalability have been shown to achieve excellent performance in many fields [31]. In the random forest algorithm, a large number of decision trees are constructed. A different subset of the data and a random selection of features are used for each decision tree to prevent overfitting in the training process. The final classification is often made by taking the majority vote. On the other hand, inherited from gradient boosting, XGBoost adds the new regression tree in each iteration to improve the previous prediction and to approach the target. The XGBoost introduces the regularization term that considers the complexity of the tree so as to avoid overfitting. In addition, the second-order gradient statistics are used for accelerating the computation.

The concept of hierarchical regression can be described as classification by random forest algorithm and then regression by XGBoost algorithm (Multimedia Appendix 3). The whole PWV range is partitioned into several subdivisions. Thus, a global classifier handles the entire PWV range, and several local regressors are in charge of the respective subdivisions. First, an outcome regarding the possible baPWV subdivision is generated by the global classifier. Then, the estimation result is calculated by the associated local regressor. Because it is possible that the data around the subdivision boundary are erroneously classified, the adjacent regressors are designed to have an overlapping zone to extend the respective coverages. Owing to the data quantity, 2 subdivisions were adopted and the boundary threshold was set at 1600 cm per second. The widths of the overlapping zone were set as 200 cm per second, 400 cm per second, and 600 cm per second.

### Statistical Analysis

The differences between the estimated results ${\hat{v}}_{j}$ and the measured PWV $v_{j}$ of the jth measurement are shown by the mean absolute error, mean error, SD, and root-mean-square error (RMSE), which are defined as follows.


(11) $$e_{j}=v_{j}-\hat{v_{j}}$$

(12) $$MAE=E\left\{\left|e_{j}\right|\right\}$$

(13) $$ME=\overset{-}{e}=E\left\{e_{j}\right\}$$

(14) $$SD=\sqrt{\frac{1}{N-1}\sum_{j=1}^{N}{\left(e_{j}-\overset{-}{e}\right)}^{2}}$$

(15) $$RMSE=\sqrt{E\left\{e_{j}^{2}\right\}}.$$

The correlation coefficients together with *P* values are also provided. Since some participants have more than 1 measurement, to avoid unbalanced weighting, averaged PWV estimation and averaged PWV measurement are used for the statistical results per participant.

## Results

In this study, 80 male participants and 82 female participants were recruited. Their demographic characteristics are shown in Table 1. The averaged PWV value of left baPWV and right baPWV was used. The PWV values of male participants and female participants were 1591 (SD 266) cm per second and 1613 (SD 321) cm per second. Among total participants, 39 male participants and 23 female participants had more than 1 PWV values due to their multiple visits. A total of 914 PPG as well as ECG sequences were collected from the smartwatch, corresponding to 278 PWV values. On average, 1 male participant has 3.5 PPG and ECG sequences associated with 1 PWV measurement while 1 female participant has 3.1 PPG and ECG sequences for 1 PWV measurement. Among 278 PWV measurements, there are 123 PWV measurements from participants taking antihypertensive medications on the same day.

The medians of the respective combined features in the 528 and 386 sequences were used for computing correlation coefficients for men and women. The correlation coefficients of combined features defined by the X and Y indices are often higher than the original one (Multimedia Appendix 4). For example, the correlation coefficients of the age and maximum slope time ($n_{ms}$) to baPWV are 0.334 and −0.281, whereas the correlation coefficient of the combined feature $Age/n_{ms}$ becomes 0.491 (Multimedia Appendix 5). The correlation coefficients of SI corrected by Friderician’s formula and the time interval between systolic peak to the onset of next PPG (P2O) normalized by pulse length from the wrist PPG versus baPWV are −0.271 (*P*<.001), −0.036 (*P*=.413) and −.370 (*P*<.001), −0.070 (*P*=.171) for men and women, respectively.

The reproducibility of the measured baPWV was also checked. The PWVs of 31 participants were measured twice by the same OMRON noninvasive vascular screening device with 1-minute separation. The maximal differences of left baPWV and right baPWV of these participants were 276 cm per second and 210 cm per second, respectively. The maximal difference of averaged baPWV from left baPWV and right baPWV was 196.5 cm per second. The RMSEs of 2 consecutively measured left baPWV and right baPWV were 83.4 cm per second and 62.0 cm per second, respectively. The RMSE of consecutive averaged baPWV was 68.8 cm per second.

For multivariable regression, 39 and 34 PWV measurements from 24 male participants and 26 female participants, respectively, were reserved as the testing dataset. The medians of the respective features from the sequences associated with the same PWV measurement were averaged. The testing dataset was selected to approach uniform distribution in the range between 1000 cm per second and 2100 cm per second. The mean and SD of the male and female PWV values in the testing dataset were 1538 (SD 237) cm per second and 1638 (SD 283) cm per second. The training dataset for deriving the coefficients contained 114 PWV measurements with 391 PPG per ECG sequences from 56 male participants and 91 PWV measurements with 291 sequences from 56 female participants. The participant-split criterion is obeyed. The baPWV estimation results by multivariable regression are shown in Table 2 for men and women, respectively.

**Table 1.** Demographic summary.^a^

Characteristics	Male participants, mean (SD; n)	Female participants, mean (SD; n)
Age (years)	63.4 (13.4; 80)	64.3 (11.6; 82)
Heart rate (bps)	73.9 (12.7; 528)	71.0 (8.2; 386)
SBP^b^ (mm Hg)	126.0 (15.7; 528)	125.9 (17.9; 386)
DBP^c^ (mm Hg)	79.4 (10.6; 528)	77.0 (12.0; 386)
PWV^d^ (cm per second)	1591 (266; 153)	1613 (321; 125)

a Among a total of 278 pulse wave velocity measurements, 123 measurements were obtained from participants taking antihypertensive medications on the same day.

b SBP: systolic blood pressure.

c DBP: diastolic blood pressure.

d PWV: pulse wave velocity.

**Table 2.** Estimation results from multivariate regression^a^.

Methods	N	MAE^b^ (cm per second)	ME^c^ (cm per second)	SD (cm per second)	RMSE^d^ (cm per second)	Correlation coefficient (*P* value)
Men						
$PWV=C_{1}Age+C_{2}\frac{SI}{M^{1/3}}+C_{3}$ [28^e^^,^^f^]	39 rounds	179.1	−49.0	214.3	217.2	0.44 (.006)
	24 participants	160.4	−40.4	195.7	195.8	0.55 (.006)
$PWV=C_{1}Age+C_{2}\frac{P2O}{M}+C_{3}$[12^g^]	39 rounds	189.0	−57.7	219.8	224.6	0.37 (.02)
	24 participants	176.1	−48.1	207.8	209.1	0.43 (.04)
Women						
$PWV=C_{1}Age+C_{2}\frac{SI}{M^{1/3}}+C_{3}$[28]	34 rounds	165.2	1.8	211.7	208.6	0.66 (<.001)
	26 participants	157.4	−12.1	197.4	194.0	0.72 (<.001)
$PWV=C_{1}Age+C_{2}\frac{P2O}{M}+C_{3}$[12]	34 rounds	196.0	10.0	233.0	229.8	0.62 (<.001)
	26 participants	188.8	8.6	221.8	217.6	0.67 (<.001)

a The testing set contained 39 and 34 pulse wave velocity measurements from 24 male participants and 26 female participants, respectively.

b MAE: mean absolute error.

c ME: mean error.

d RMSE: root-mean-square error.

e PWV indicates pulse wave velocity.

f SI: stiffness index.

g P2O: systolic peak to the next onset.

For hierarchical regression, the same training and testing datasets as those in multivariable regression were used to keep participants split. The training dataset was oversampled to make the distribution balanced in each interval of 100 cm per second. Several parameters, such as the shrinkage factor, tree depth, and column subsampling, are required for the random forest and XGBoost algorithms. Hence, a validation set split from the training dataset was used for parameter settings. Because the number of PWV measurements of extreme high and low values was not sufficiently large, leave-one-out validation was used to ensure that the model for validation is similar to that for training. For the general model, the male validation set contained 23 participants and 33 PWV measurements, while the female validation set had 22 participants and 39 PWV measurements. The validation set consisted of more than one-third of participants in the training dataset and kept uniformly distributed in the range from 1000 cm per second to 2100 cm per second. During leave-one-out validation, all the PPG or ECG sequences associated with the PWV measurements of 1 validation participant were removed from the training dataset to avoid data leak. For each submodel of the local regressor, the validation dataset in each subdivision includes those with the PWV measurements in the overlapping zone. Given the overlapping zone of 400 cm per second, there were 24 PWV measurements from 13 male participants and 26 PWV measurements from 12 female participants in the high submodel for validation from 1400 cm per second. On the other hand, 25 PWV measurements from 13 male participants and 25 PWV measurements from 16 female participants were used in the low submodel for validation up to 1800 cm per second.

**Figure 5.**
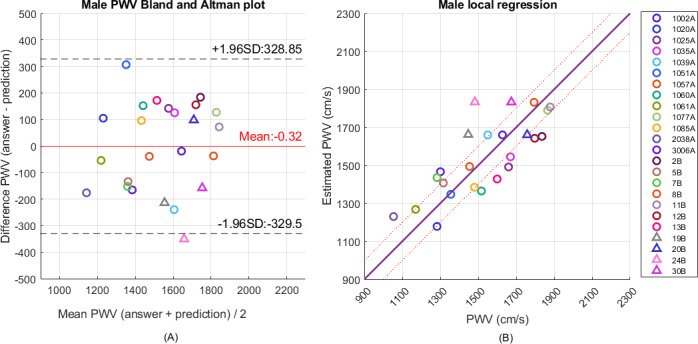
(A) Bland-Altman plot and (B) scatter plot of pulse wave velocity regression by the hierarchical regression model with 2 submodels and overlapping zone of 400 cm per second for 24 men. PWV: pulse wave velocity.

Table 3 lists the estimation results from the general and hierarchical regression models by the random forest classification and XGBoost regression algorithms with different settings of the width of the overlapping zones. First, the RMSE results from the hierarchical regression models are better than those from the multivariable linear regression model. The hierarchical regression model also outperforms the general regression model. Figures5  6 show the Bland-Altman and scatter plots of regression results by the hierarchical regression model with overlapping zone of 400 cm per second for men and women participants. Their participant numbers are indicated in the legend. Good estimation was obtained for this setting. The left subfigures indicate the Bland-Altman plot. The scatter plots in the right subfigures provide the final estimation results. The classification accuracies of total rounds from male participants and female participants are 76.9% and 91.2%, respectively. The estimation of erroneously classified data close to the boundary got improved with the introduction of an overlapping zone. The best estimation results achieve RMSE of 145.0 cm per second and 141.4 cm per second for men and women, respectively. In the random forest classifier for male participants, the number of estimators is 100 and the maximum tree depth is 20. As to the random forest for female participants, the number of estimators is 250 and the maximum tree depth is 9. In both cases, the minimum samples for tree split should be larger than 2 and the minimum number of samples in leaf nodes is 1. As to the XGBoost regressors, the number of estimators is 200; the fraction of features sampled for each tree is 0.7; and the minimum loss reduction for further partition is 0. The maximum depth of the low submodel for male participants is 5 and is set to 3 for the remaining submodels.

The XGBoost algorithm performs tree splitting by evaluating structure scores to accumulate gradient statistics according to the sorted feature values while the random forest algorithm can assess the impact on pureness of the leaves from a feature. Hence, both can report the feature importance. Given the overlapping zone of 400 cm per second in the hierarchical regression model, besides PAT ($n_{R}$), PAT square ($n_{R}^{2}$), and age, PPG features and WPD features were also frequently used (Multimedia Appendix 6). Local regression models used features different from those used in global classification models. Features from component wave, points a, b, c, and d of SDPPG were often adopted.

**Table 3.** Hierarchical regression results for men and for women are listed.

Method	Overlapping zone (cm per second)	N	MAE^a^ (cm per second)	ME^b^ (cm per second)	SD (cm per second)	RMSE^c^ (cm per second)	Correlation coefficient (*P* value)
Men							
General regression	—^d^	39 rounds	157.4	−16.5	187.0	185.3	0.61 (<.001)
General regression	—	24 participants	141.7	−8.4	173.1	169.7	0.66 (<.001)
Hierarchical regression	200	39 rounds	156.0	−19.4	185.3	183.9	0.64 (<.001)
Hierarchical regression	200	24 participants	152.1	−18.4	185.6	182.6	0.63 (.001)
Hierarchical regression	400	39 rounds	133.6	−8.1	160.1	158.3	0.74 (<.001)
Hierarchical regression	400	24 participants	126.5	−8.9	147.8	*145.0* ^e^	*0.77*^e^ (<.001)
Hierarchical regression	600	39 rounds	153.6	−2.3	182.9	180.5	0.63 (<.001)
Hierarchical regression	600	24 participants	143.6	13.7	165.0	162.1	0.70 (<.001)
Women							
General regression	—	34 rounds	174.3	−36.0	217.0	216.8	0.67 (<.001)
General regression	—	26 participants	177.7	−22.4	217.8	214.7	0.66 (<.001)
Hierarchical regression	200	34 rounds	141.5	−20.7	171.0	169.7	0.80 (<.001)
Hierarchical regression	200	26 participants	131.4	−29.2	157.4	157.0	0.83 (<.001)
Hierarchical regression	400	34 rounds	127.3	−3.5	156.7	154.5	0.83 (<.001)
Hierarchical regression	400	26 participants	116.7	−6.0	144.1	*141.4* ^e^	*0.86*^e^ (<.001)
Hierarchical regression	600	34 rounds	144.3	24.2	173.9	173.0	0.79 (<.001)
Hierarchical regression	600	26 participants	141.2	24.0	173.5	171.8	0.79 (<.001)

a MAE:mean absolute error.

b ME: mean error.

c RMSE: root-mean-square error.

d Not applicable.

e Values in italics indicate best estimation result with acceptable accuracy set by the ARTERY Society.

**Figure 6.**
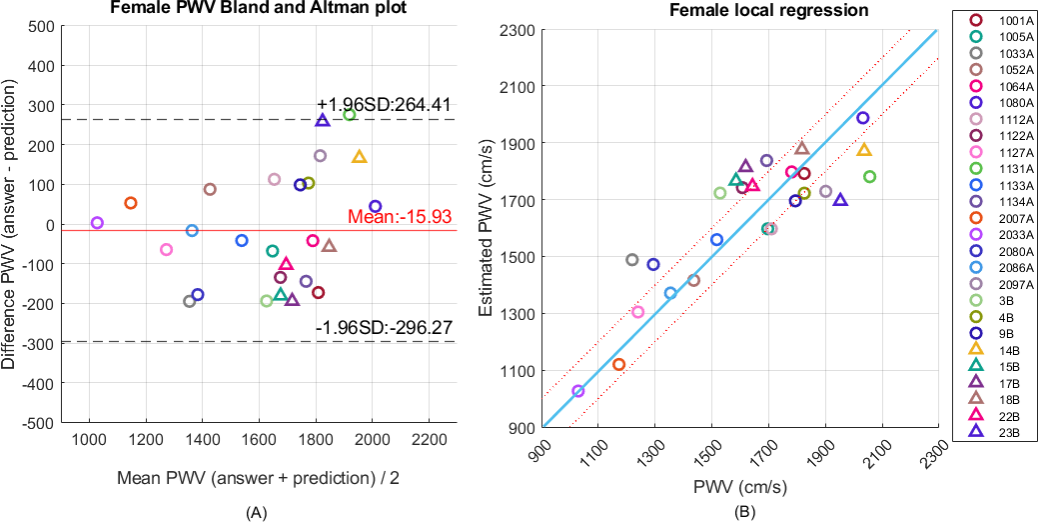
(A) Bland-Altman plot and (B) scatter plot of pulse wave velocity regression by the hierarchical regression model with 2 submodels and overlapping zone of 400 cm per second for 26 women. PWV: pulse wave velocity.

## Discussion

### Principal Findings

In this study, we used wrist PPG and ECG signals to estimate baPWV. The morphology of wrist PPG signals is quite different from that of finger PPG signals. The conventional approach that used finger PPG morphology features may encounter the problem of feature missing due to much fewer identifiable features of wrist PPG signals. In addition, the multivariable regression model used in prior works may be too simple to describe the complicated hemodynamic state in the vessels. Hence, we resorted to the machine learning algorithm to deal with the estimation. Although the wrist PPG and ECG signals were acquired before the baPWV measurement, they are still related to the vessel condition and stiffness. To further improve and refine the estimation results, hierarchical regression was adopted to shrink the range handled in the submodel. The achieved RMSE and SD by our hierarchical regression models for both men and women are lower than the threshold (150 cm per second) of acceptable accuracy for PWV estimation set by the ARTERY Society [32].

### Comparison With Prior Work

With the WPD and feature imputation techniques developed by us, more than 98% of all ambiguous and missing features of wrist PPG can be identified [19]. From the correlation results (Multimedia Appendix 4), besides age (feature 23) and age square (feature 63), correlation related to SDPPG amplitude of point c (feature 18), point d (feature 19), and point e (feature 20) are still obvious as what has been mentioned in finger PPG [25]. In addition, SI ($n_{p_{d}}-n_{p_{s}}$; feature 51), which are often missing in the original wrist PPG pulses, can be computed through the synthesized systolic and diastolic waves in decomposed wrist PPG. According to the feature importance (Multimedia Appendix 6), it still plays an important role for PWV estimation.

The multivariable regression uses only a few features. If significantly high correlations of those features to baPWV do not appear, the performance of estimation will be degraded. However, the machine learning algorithm can help exploit more linear or nonlinear information embedded in the PPG waveform or its component waves and thus is suitable for these applications. Furthermore, the combined features from PPG and ECG morphology, WPD, and basic information supplied more feature information sources that can be selected by the model.

### Hierarchical Model Insights

The concept of hierarchical regression is to introduce different models to refine the estimation results. However, the global classifier or regressor must provide sufficiently correct classification to avoid model mismatch. From the hierarchical regression results, it is clear that the inclusion of overlapping zone in local regressors indeed improved the estimation results, as reflected in the improved correlation coefficients (Table 2). However, the determination of optimal range of overlapping zone is still controversial. If the overlapping zone is too wide, the hierarchical regression model would become similar to the general regression model. On the other hand, if the overlapping zone is too narrow, the misclassified data cannot be properly handled. In this study, we recommend the overlapping zone of 400 cm per second of 2 subdivision models because the misclassified data are near the boundary due to good capability of the global classifier and can be appropriately covered by the submodel. We conducted further analysis on the features that were misclassified for those samples not near the decision boundary. The results showed no significant outliers. Additionally, the vote counts for 2 classes across the entire forest were close, indicating low confidence among the trees. The latent properties beyond the observed features should be further studied. On the other hand, we also applied a Kernel Density Estimation–based mutual information analysis [33] to assess the relevance of individual features in male and female datasets. The mutual information values from male features were lower than those from female features, which can also explain the lower classification accuracy in male participants of our dataset.

### Limitations and Future Directions

This study has limitations, which point to the directions for future research. First, the sample size remained small and more older adult people were recruited in the study, which might limit its applicability in younger populations. While the current dataset demonstrates feasibility in estimating PWV using wrist PPG in older individuals, the skewed dataset toward older individuals may have influenced the performance due to age-related vascular characteristics. In future work, we plan to expand the study population by actively recruiting more young participants. The inclusion of younger participants will help balance the age distribution and allow for more robust assessment of the model performance across different age groups. This extension will not only improve the generalizability of the model but also enable a more comprehensive evaluation of age-related vascular changes. Second, the current model adopts machine learning algorithms to exploit linear and nonlinear features within the scope of this dataset. As the dataset grows in size and diversity, other deep learning algorithms, such as Bayesian neural networks or multilayer perceptrons, can be applied, which may offer better uncertainty quantification or modeling capabilities. Third, the feature space used in the current model is relatively high-dimensional, which may hinder its practical deployment on wearable or edge devices with limited computational resources. Feature compression or dimensionality reduction techniques can be considered to decrease model complexity in the future. This optimization will help make the system more suitable for real-time, low-power applications in wearable health care settings. Together, these improvements aim to enhance both the robustness and the applicability of the proposed approach, facilitating its transition toward practical use in diverse and real-world scenarios.

## Supplementary material

10.2196/58756 Multimedia Appendix 1 (A) Electrocardiography before preprocessing, (B) photoplethysmography before preprocessing, (C) electrocardiography with R peak after preprocessing, and (D) photoplethysmography with valley after preprocessing.

10.2196/58756 Multimedia Appendix 2 List of extracted features: (A) photoplethysmography features, (B) weighted pulse decomposition features, (C) electrocardiography features, and (D) basic information.

10.2196/58756 Multimedia Appendix 3 Concept of hierarchical regression.

10.2196/58756 Multimedia Appendix 4 Heat map of correlation coefficients of combined features (defined in Tables S1 A, S1 B, S1 C, and S1 D in Multimedia Appendix 2) versus brachial-ankle pulse wave velocity for (A) 80 male participants and (B) 82 female participants with 528 and 386 data, respectively. The diagonal elements are the correlation coefficients of original features. The off-diagonal elements are the correlation coefficients of combined features.

10.2196/58756 Multimedia Appendix 5 The distributions of brachial-ankle pulse wave velocity versus (A) age, (B) n_ms_, and (C) Age/n_ms_ for 386 female data.

10.2196/58756 Multimedia Appendix 6 Top 10 important features of classification and local regression for (A) men and (B) women.

## Abbreviations

baPWC: brachial-ankle pulse wave velocity
cfPWV: carotid-femoral pulse wave velocity
CVD: cardiovascular disease
ECG: electrocardiography
FDPPG: first-order derivative photoplethysmography
PAT: pulse arrival time
PPG: photoplethysmography
P2O: peak to the next onset
PWV: pulse wave velocity
RMSE: root-mean-square error
SDPPG: second-order derivative photoplethysmography
SI: stiffness index
WPD: weighted pulse decomposition
XGBoost: extreme gradient boosting
