# Supplementary figures and images for: Estimation of Brachial-Ankle Pulse Wave Velocity With Hierarchical Regression Model From Wrist Photoplethysmography and Electrocardiographic Signals: Method Design
Source: JMIR Biomed Eng. 2025 Aug 26;10:e58756. doi: 10.2196/58756 (PMC12423722; doi:10.2196/58756)

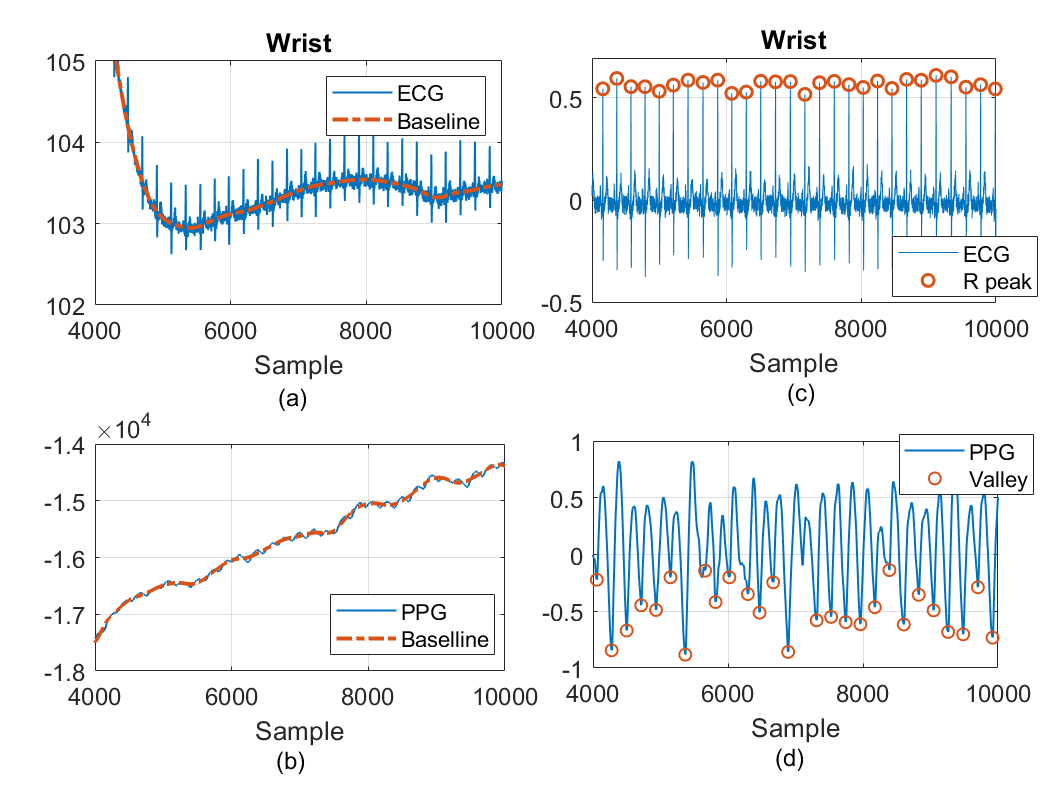

Supplement: Multimedia Appendix 1 [file biomedeng-v10-e58756-s001.png]

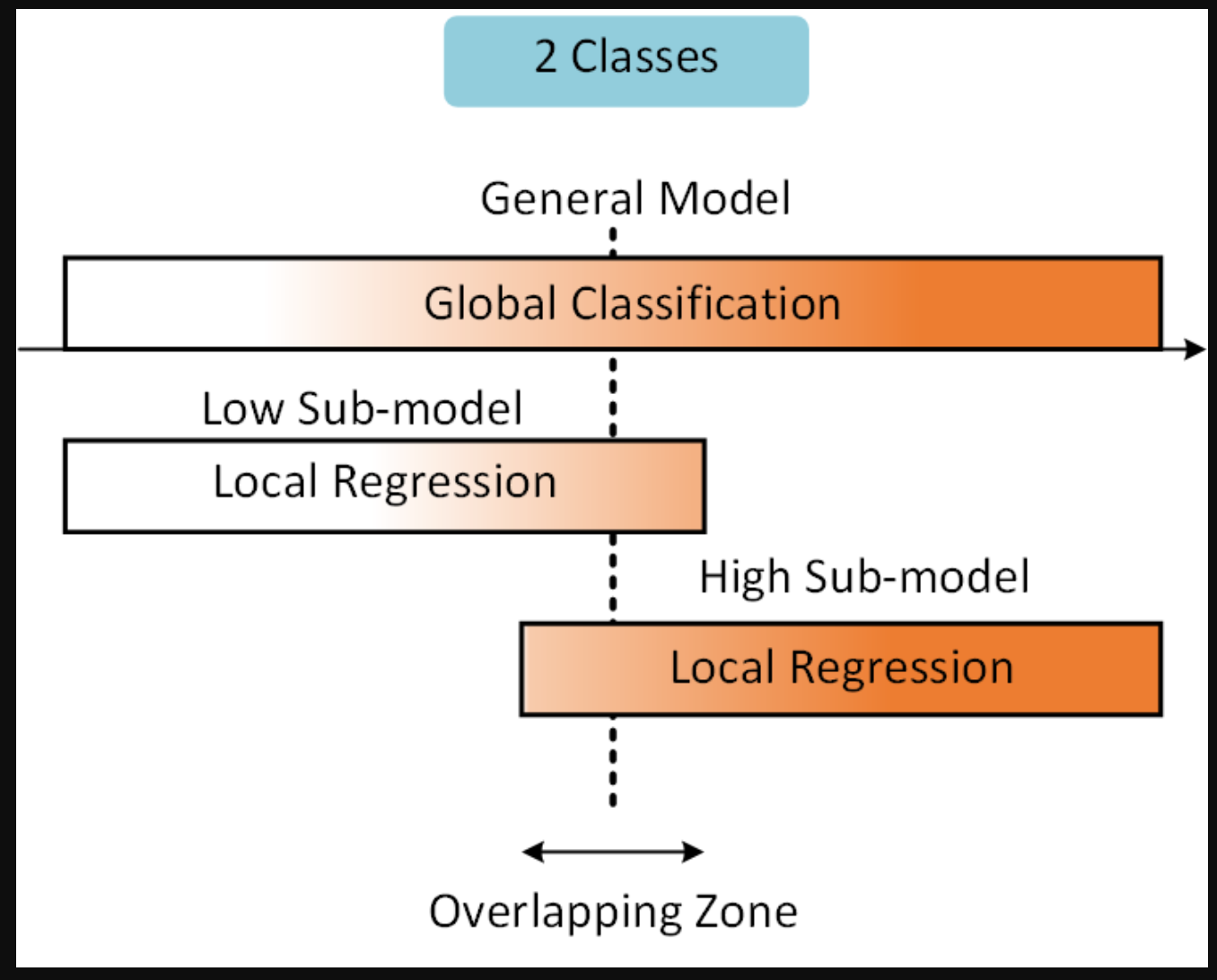

Supplement: Multimedia Appendix 3 [file biomedeng-v10-e58756-s003.png]

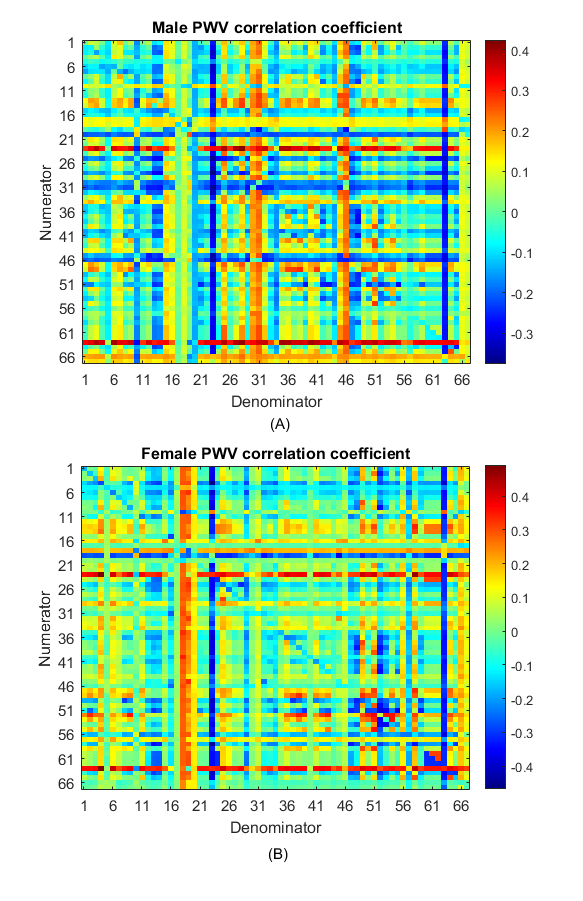

Supplement: Multimedia Appendix 4 [file biomedeng-v10-e58756-s004.png]

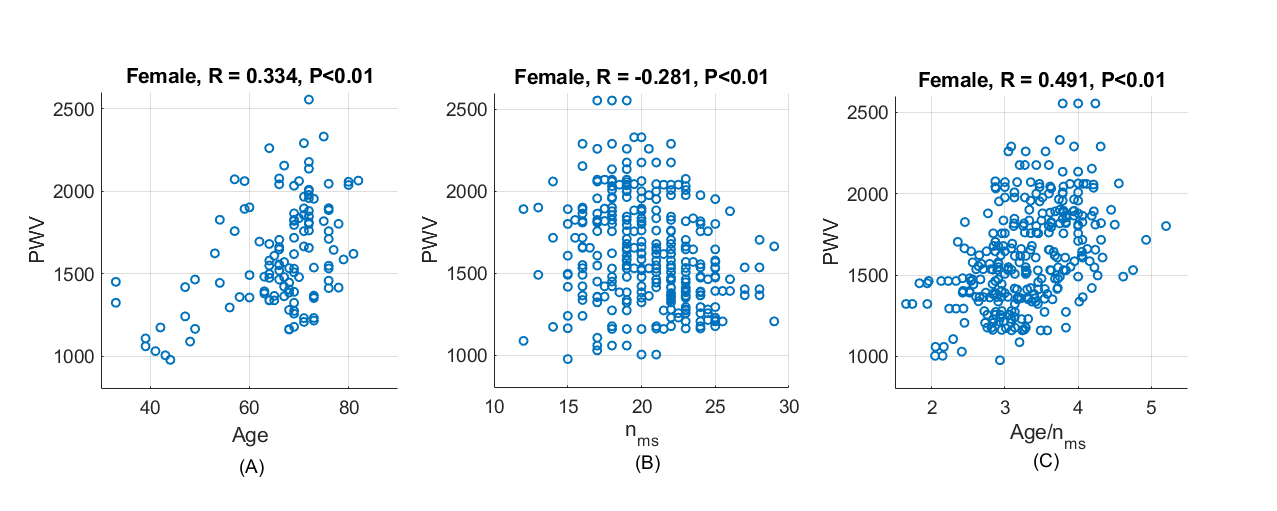

Supplement: Multimedia Appendix 5 [file biomedeng-v10-e58756-s005.png]
