# Supplementary material for: Estimation of Brachial-Ankle Pulse Wave Velocity With Hierarchical Regression Model From Wrist Photoplethysmography and Electrocardiographic Signals: Method Design
Source: JMIR Biomed Eng. 2025 Aug 26;10:e58756. doi: 10.2196/58756 (PMC12423722; doi:10.2196/58756)
Supplement: Multimedia Appendix 2 [file biomedeng-v10-e58756-s002.docx]

**Multimedia Appendix 2 Feature Table S1**

1. PPG features

| Index | Feature | Index | Feature |
| --- | --- | --- | --- |
| 1 | Systolic Peak$(n_{sys})$ | 2 | Diastolic Peak $(n_{dia})$ |
| 3 | Notch $(n_{not})$ | 4 | Max Slope $(n_{ms})$ |
| 5 | Point a $(n_{a})$ | 6 | Point b $(n_{b})$ |
| 7 | Point c $(n_{c})$ | 8 | Point d $(n_{d})$ |
| 9 | Point e $(n_{e})$ | 10 | Skewness |
| 12 | Systolic Peak $(A_{sys})$ | 13 | Diastolic Peak $(A_{dia})$ |
| 14 | Notch $(A_{not})$ | 15 | Max Slope $(A_{ms}^{(1)})$ |
| 16 | Point a $(A_{a}^{(2)})$ | 17 | Point b $(A_{b}^{(2)})$ |
| 18 | Point c $(A_{c}^{(2)})$ | 19 | Point d $(A_{d}^{(2)})$ |
| 20 | Point e $(A_{e}^{(2)})$ | 21 | Pulse Length $(M)$ |
| 66 | Age Index | 67 | $(A_{b}^{\left( 2 \right)}-A_{c}^{\left( 2 \right)}-A_{d}^{\left( 2 \right)})/A_{a}^{\left( 2 \right)}$ |

1. WPD features

| Index | Feature | Index | Feature |
| --- | --- | --- | --- |
| 30 | Component Wave 1 $(\alpha_{1})$ | 31 | Component Wave 2 $(\alpha_{2})$ |
| 32 | Component Wave 3 $(\alpha_{3})$ | 33 | Component Wave 4 $(\alpha_{4})$ |
| 34 | Component Wave 5 $(\alpha_{5})$ | 35 | Component Wave 1 $(\beta_{1})$ |
| 36 | Component Wave 2 $(\beta_{2})$ | 37 | Component Wave 3 $(\beta_{3})$ |
| 38 | Component Wave 4 $(\beta_{4})$ | 39 | Component Wave 5 $(\beta_{5})$ |
| 40 | Component Wave 1 $(\gamma_{1})$ | 41 | Component Wave 2 $(\gamma_{2})$ |
| 42 | Component Wave 3 $(\gamma_{3})$ | 43 | Component Wave 4 $(\gamma_{4})$ |
| 44 | Component Wave 5 $(\gamma_{5})$ | 45 | Systolic Wave $(A_{p_{s}})$ |
| 46 | Forward Wave $(A_{p_{f}})$ | 47 | Diastolic Wave $(A_{p_{d}})$ |
| 48 | Systolic Wave $(n_{p_{s}})$ | 49 | Forward Wave $(n_{p_{f}})$ |
| 50 | Diastolic Wave $(n_{p_{d}})$ | 51 | SI $(n_{p_{d}}-n_{p_{s}})$ |
| 52 | $(\beta_{3}-n_{p_{f}})$ | 53 | $(\beta_{4}-n_{p_{f}})$ |
| 54 | $(\beta_{4}-\beta_{3})$ | 55 | $(n_{p_{d}}-n_{p_{f}})$ |
| 59 | $(n_{p_{d}}-n_{ms})$ |  |  |

(c) ECG features

| Index | Feature | Index | Feature |
| --- | --- | --- | --- |
| 11 | PAT $(-n_{R})$ | 22 | T Peak to R Peak $(n_{T}-n_{R})$ |
| 29 | Height/PAT $(-H/n_{R})$ | 56 | PAT^2^ $(n_{R}^{2})$ |
| 57 | Height^2^/PAT^2^ $(H^{2}/n_{R}^{2})$ | 58 | R Peak to max Slope $(n_{ms}-n_{R})$ |
| 64 | $(\beta_{2}-n_{R})$ | 65 | $(n_{p_{s}}-n_{R})$ |

(d) Basic Information

| Index | Feature | Index | Feature |
| --- | --- | --- | --- |
| 23 | Age | 24 | Height $(H)$ |
| 25 | Weight $(W)$ | 26 | $L_{aw}$ |
| 27 | $L_{af}$ | 28 | BMI |
| 63 | Age^2^ | 60 | $L_{b}$ |
| 61 | $L_{a}$ | 62 | $L_{a}-L_{b}$ |
