# Supplementary material for: Estimation of Brachial-Ankle Pulse Wave Velocity With Hierarchical Regression Model From Wrist Photoplethysmography and Electrocardiographic Signals: Method Design
Source: JMIR Biomed Eng. 2025 Aug 26;10:e58756. doi: 10.2196/58756 (PMC12423722; doi:10.2196/58756)
Supplement: Multimedia Appendix 6 [file biomedeng-v10-e58756-s006.docx]

**Multimedia Appendix 6**

**Top 10 important features of classification and local regression for men and women.**

1. Men

| Classification | | Regression (Low Sub-model) | | Regression (High Sub-model) | |
| --- | --- | --- | --- | --- | --- |
| Numerator | Denominator | Numerator | Denominator | Numerator | Denominator |
| $L_{a}-L_{b}$ | $(\beta_{2}-n_{R})$ | Age^2^ | Height | $L_{a}$ | Age^2^ |
| Age^2^ | $L_{af}$ | $\beta_{4}-\beta_{3}$ | $H^{2}/n_{R}^{2}$ | $L_{b}$ | Age^2^ |
| $L_{a}$ | $(\beta_{2}-n_{R})$ | $L_{aw}$ | $\alpha_{2}$ | $A_{not}$ | Age^2^ |
| Age^2^ | Weight | $n_{ms}-n_{R}$ | | $n_{p_{d}}-n_{p_{s}}$ | $n_{sys}$ |
| $L_{aw}$ | Age^2^ | $A_{sys}$ | Age | $n_{d}$ | $A_{p_{s}}$ |
| $L_{a}$ | BMI | $N$ | $n_{b}$ | $\gamma_{1}$ | $n_{e}$ |
| Age^2^ | $A_{dia}$ | $\alpha_{3}$ | Age | $n_{p_{d}}-n_{p_{s}}$ | $\beta_{4}-\beta_{3}$ |
| $L_{aw}$ | Height | $A_{b}^{(2)}$ | $L_{aw}$ | Weight | Height |
| $A_{dia}$ | $(\beta_{2}-n_{R})$ | $n_{c}$ | $A_{p_{d}}$ | $n_{b}$ | $n_{p_{s}}-n_{R}$ |
| $L_{aw}$ | Weight | Age^2^ | $A_{e}^{(2)}$ | Height | $L_{a}-L_{b}$ |

(b) Women

| Classification | | Regression (Low Sub-model) | | Regression (High Sub-model) | |
| --- | --- | --- | --- | --- | --- |
| Numerator | Denominator | Numerator | Denominator | Numerator | Denominator |
| $n_{c}$ | Height | $L_{aw}$ | $\beta_{2}$ | $n_{p_{s}}$ | $\beta_{3}-n_{p_{f}}$ |
| $\gamma_{4}$ | Height | Age | $n_{c}$ | $L_{aw}$ | Age |
| $\beta_{1}$ | Height | skewness | $L_{aw}$ | $n_{c}$ | Weight |
| Age^2^ | Weight | $A_{a}^{(2)}$ | $A_{c}^{(2)}$ | $L_{aw}$ | |
| Height | Age^2^ | $n_{d}$ | $n_{T}-n_{R}$ | $\beta_{5}$ | $n_{ms}$ |
| $n_{p_{d}}-n_{ms}$ | $(\beta_{2}-n_{R})$ | $n_{T}-n_{R}$ | $n_{not}$ | $\beta_{4}$ | $A_{dia}$ |
| Age^2^ | Age | $\beta_{1}$ | $A_{dia}$ | $L_{aw}$ | $\gamma_{1}$ |
| Age^2^ | $n_{p_{f}}$ | $n_{p_{d}}-n_{ms}$ | $\beta_{2}$ | $A_{ms}^{(1)}$ | $A_{a}^{(2)}$ |
| $A_{p_{d}}$ | $(\beta_{3}-n_{p_{f}})$ | $\alpha_{3}$ | $\alpha_{4}$ | $n_{p_{d}}-n_{p_{s}}$ | $\gamma_{4}$ |
| $\gamma_{1}$ | $(\beta_{2}-n_{R})$ | $\alpha_{3}$ | $-n_{R}$ | $L_{aw}$ | $n_{p_{d}}-n_{ms}$ |
